# Characterization of Cognitive Deficits in Mice With an Alternating Hemiplegia-Linked Mutation

**DOI:** 10.1037/bne0000097

**Published:** 2015-10-26

**Authors:** Greer S. Kirshenbaum, James Dachtler, John C. Roder, Steven J. Clapcote

**Affiliations:** 1 Lunenfeld-Tanenbaum Research Institute, Mount Sinai Hospital, Toronto, Ontario, and Institute of Medical Science, University of Toronto; 2 School of Biomedical Sciences, University of Leeds; 3 Lunenfeld-Tanenbaum Research Institute, Mount Sinai Hospital, Toronto, Ontario, and Institute of Medical Science, University of Toronto; 4 School of Biomedical Sciences, University of Leeds

**Keywords:** cognitive deficits, Na^+^,K^+^-ATPase α3, *Atp1a3*, mice, alternating hemiplegia

## Abstract

Cognitive impairment is a prominent feature in a range of different movement disorders. Children with Alternating Hemiplegia of Childhood are prone to developmental delay, with deficits in cognitive functioning becoming progressively more evident as they grow older. Heterozygous mutations of the *ATP1A3* gene, encoding the Na^+^,K^+^-ATPase α3 subunit, have been identified as the primary cause of Alternating Hemiplegia. Heterozygous *Myshkin* mice have an amino acid change (I810N) in Na^+^,K^+^-ATPase α3 that is also found in Alternating Hemiplegia. To investigate whether *Myshkin* mice exhibit learning and memory deficits resembling the cognitive impairments of patients with Alternating Hemiplegia, we subjected them to a range of behavioral tests that interrogate various cognitive domains. *Myshkin* mice showed impairments in spatial memory, spatial habituation, locomotor habituation, object recognition, social recognition, and trace fear conditioning, as well as in the visible platform version of the Morris water maze. Increasing the duration of training ameliorated the deficit in social recognition but not in spatial habituation. The deficits of *Myshkin* mice in all of the learning and memory tests used are consistent with the cognitive impairment of the vast majority of AHC patients. These mice could thus help advance our understanding of the underlying neural mechanisms influencing cognitive impairment in patients with *ATP1A3*-related disorders.

Cognitive impairment is a prominent feature in a range of different movement disorders, with a clear cognitive phenotype coexisting alongside the motor abnormality (Walterfang & van de Warrenburg, 2014). Children with a rare neurodevelopmental disorder, Alternating Hemiplegia of Childhood (AHC; OMIM: 614820), are prone to developmental delay, with deficits in cognitive functioning becoming progressively more evident as they grow older (Panagiotakaki et al., 2010; Sweney et al., 2009). In two large studies, a variable degree of cognitive impairment was observed as a nonepisodic symptom in 94% (144 of 153; Panagiotakaki et al., 2010) and 100% (96 of 96; Sweney et al., 2009) of AHC patients surveyed. Among 144 AHC patients with cognitive impairment, Panagiotakaki et al. (2010) reported that the intellectual disability was mild (IQ 50–70) in 20%, moderate (IQ 35–50) in 54%, and severe (IQ 20–35) in 26%. Neuropsychological evaluation of 41 affected individuals by Sweney et al. (2009) revealed an average full-scale IQ of 62.5 ± 14.0 (mild intellectual disability), with less impairment of cognitive and adaptive skills in younger patients. Additional deficits in speech, attention, psychomotor abilities, and psychosocial functioning have also been noted (Neville & Ninan, 2007; Shafer, Mayfield, & McDonald, 2005). Flunarizine, a calcium channel blocker, is reported to alleviate the frequency and severity of hemiplegia in some patients, but there is no evidence that it has any effect on the persistent cognitive deficits that add to the burden of disease (Silver & Andermann, 1993).

Heterozygous mutations of the *ATP1A3* gene, encoding the Na^+^,K^+^-ATPase α3 subunit, have been identified as the primary cause of AHC (Heinzen et al., 2014). Na^+^,K^+^-ATPases are membrane-bound transporters that harness the energy of ATP hydrolysis to pump three Na^+^ ions out of the cell in exchange for two K^+^ ions moving inward. The main role of the α-subunit is to bind and transport Na^+^ and K^+^ (Benarroch, 2011). The Na^+^,K^+^-ATPase α3 subunit is exclusively present in neurons, where it is thought to be responsible for restoration of basal intracellular Na^+^ concentrations after sustained activity (Azarias et al., 2013). This Na^+^,K^+^-ATPase consumes about 50% of the energy of neurons and is vulnerable to metabolic stress (Bøttger et al., 2011). Na^+^,K^+^-ATPase α3 immunofluorescence is found preferentially localized to γ-aminobutyric acid (GABA)ergic neurons in human and rodent brains (Bøttger et al., 2011; Paciorkowski et al., 2015). To date, 59 different de novo missense mutations in *ATP1A3* have been identified in AHC patients, including three affecting the isoleucine at position 810: I810F, I810N, and I810S (Heinzen et al., 2014; Panagiotakaki et al., 2015; Rosewich, Ohlenbusch et al., 2014; Sasaki et al., 2014; Ulate-Campos et al., 2014; Viollet et al., 2015; Yang et al., 2014).

Twelve other missense mutations in *ATP1A3* have been identified in patients diagnosed with rapid-onset dystonia-parkinsonism (RDP; DYT12; OMIM: 128235), a movement disorder characterized by abrupt onset of the permanent symptoms of dystonia with parkinsonism, often after a stressful event, typically in late adolescence or early adulthood (Heinzen et al., 2014). As with other forms of dystonia (Stamelou, Edwards, Hallett, & Bhatia, 2012; Walterfang & van de Warrenburg, 2014), there is growing evidence for an important nonmotor component to RDP (Barbano et al., 2012). Cognitive impairments in verbal fluency and executive functioning tasks have been exhibited by RDP patients and an asymptomatic relative, all carrying a T613M mutation (Barbano et al., 2012). Three RDP mutations and one additional mutation have been identified in patients with an intermediate AHC/RDP presentation (Boelman et al., 2014; Heinzen et al., 2014; Rosewich et al., 2014; Roubergue et al., 2013; Sasaki, Ishii, Saito, & Hirose, 2014). A further missense mutation (E818K) has been identified in patients with CAPOS syndrome (*C*erebellar ataxia with *A*reflexia, *P*es cavus, *O*ptic atrophy, and *S*ensorineural deafness; OMIM: 601338; Demos et al., 2014; Heimer et al., 2015), and in a patient with an intermediate CAPOS/AHC presentation (Rosewich, Weise et al., 2014).

During seizures, there is often an energy crisis in neurons, which can lead to Na^+^,K^+^-ATPase pump failure (Araujo et al., 2014). Such failure could influence the pathophysiology of seizure activity of any etiology, as well as contribute to its long-term consequences and possibly even to sudden unexpected death in epilepsy (SUDEP; Richter et al., 2012). Indeed, two further missense mutations in *ATP1A3* have been found in a child with catastrophic early life epilepsy (G358V), and in another child with epilepsy, episodic prolonged apnea, postnatal microcephaly, and severe developmental disability (I363N; Paciorkowski et al., 2015).

Heterozygous *Myshkin* (*Atp1a3*^*Myk*/+^; *Myk*/+) mutant mice have an I810N amino acid substitution in transmembrane α-helix TM6 that is identical to that present in AHC (Clapcote et al., 2009; Panagiotakaki et al., 2015; Yang et al., 2014). I810N results in a normally expressed, but inactive, α3 protein and a subsequent 42% reduction of total Na^+^,K^+^-ATPase activity in the mouse brain (Clapcote et al., 2009). Molecular modeling of I810N has shown that it brings about severe structural effects on Na^+^,K^+^-ATPase α3, including the capacity for efficient K^+^ movement along the K^+^ access pathway (Kirshenbaum et al., 2013). We previously reported that *Myk*/+ mice show deficits in contextual fear conditioning, delay cued fear conditioning, and conditioned taste aversion tests of learning and memory (Kirshenbaum et al., 2013), but their normal head tracking in an optokinetic drum suggests that their vision is not impaired (Kirshenbaum, Clapcote et al., 2011). These mice also show deficits in both frontal cortex functioning (hypofrontality) and thalamocortical functional connectivity, as revealed by glucose metabolism imaging (Kirshenbaum et al., 2013). However, hippocampal slices from *Myk*/+ mice showed wild-type-like levels of θ burst stimulation-evoked long-term potentiation (LTP; Clapcote et al., 2009), a persistent increase in synaptic strength that is widely considered one of the major cellular mechanisms that underlies learning and memory (Cooke & Bliss, 2006). The purpose of the present study was to extend our phenotypic characterization of cognitive functioning in *Myk*/+ mice, by subjecting them to a range of behavioral tests that interrogate various cognitive domains.

## Method

### Subjects

*Myk/+* mice have an amino acid change (I810N) that was generated through *N-*nitroso-*N*-ethylurea (ENU) mutagenesis (Clapcote et al., 2009). *Myk*/+ males, backcrossed for 20 generations to the C57BL/6NCr strain (NCI-Frederick), were mated with C57BL/6NCrl (Charles River) females to yield wild-type (+/+) and *Myk*/+ littermates. *Myk*/+ mice were genotyped by the presence of an EcoO109I (New England BioLabs) restriction site using polymerase chain reaction primers F, 5′-CTG CCG GAA ATA CAA TAC TGA-3′ and R, 5′-ATA AAT ACC CCA CCA CTG AGC-3′. Mice were group housed (2–5 mice/cage) with same-sex littermates.

### Behavioral Procedures

*Myk*/+ and +/+ littermates were tested at 8–14 weeks of age in four cohorts. Males and females were included in balanced numbers, apart from in the social recognition and open field tests. Subjects were handled daily for 5 min/day for 7 days before behavioral testing. Experimenters were blinded to genotype during behavioral testing. Behavioral testing apparatus were cleaned with 70% ethanol between subjects. The order of tests, with a rest period of 3–5 days between each test, was as follows: Cohort 1 (*n* = 10/genotype): Y maze → object recognition → T maze (10-min); Cohort 2 (*n* = 10/genotype): T maze (1 hr) → Morris water maze → trace fear conditioning; Cohort 3 (*n* = 9/genotype; males only): social recognition (2-min); Cohort 4 (*n* = 6/genotype; males only): social recognition (10-min) → open field. All procedures were approved by the University of Leeds Ethical Review Committee and were conducted in accordance with the requirements of the Animals (Scientific Procedures) Act, 1986.

#### Y-maze spontaneous alternation

The Y-maze spontaneous alternation test is based on the natural tendency of rodents to explore a novel environment. The Y-maze apparatus made of matt white acrylic sheet had three identical arms (35 × 5 × 15 cm) placed at 120° from each other. Each mouse received one trial, in the course of which the subject was placed into one of the three arms and allowed free exploration of the maze for 5 min. The number of arm entries and the number of alternations were recorded to calculate the percentage of alternation. Over the course of multiple arm entries, mice typically exhibit a tendency to explore the least recently visited arm, and thus, tend to alternate visits between the three arms. For efficient alternation, mice need to use working memory, and thus, they should maintain an ongoing record of most recently visited arms, and continuously update such a record. A mouse with an impaired working memory cannot remember which arm it has just visited, and thus shows decreased spontaneous alternation (King & Arendash, 2002). Alternation was defined as the number of arm choices that differed from the previous two choices. The alternation percentage was calculated as: “number of alternations” divided by “total number of arm entries” minus 2. Thus, if a subject made the following sequence of arm choices (3, 2, 1, 2, 3, 2, 1, 3), the total number of alternation opportunities would be six (total entries minus 2) and the percentage alternation would be 67% (4 of 6).

#### Object recognition

The object recognition test was conducted as previously described (Ricceri, Colozza, & Calamandrei, 2000). The test exploits the natural exploratory activity of rodents toward spatial novelty to assess the detection of spatial relocation of a known object and is critically dependent on the hippocampus (Stupien, Florian, & Roullet, 2003). Mice were placed into a testing arena consisting of an open field made of clear acrylic sheet (41.25 × 41.25 × 31.25 cm) in dim light. After 10-min of habituation, mice were removed from the arena and returned to the home cage. Four identical objects (1–4; glass Petri dishes) were placed into the arena, each in a separate corner 4-cm away from the wall. After 2 min, mice were returned to the arena, and left to explore for 15 min. Mice were then removed from the arena and returned to the home cage. Two of the objects (3 and 4) in the arena were then displaced from their original locations. After 2 min, mice were placed back into the arena, and left to explore for 5 min. Mice were then removed from the arena and returned to the home cage. Three of the original objects (2–4) were then removed from the arena and replaced with a single novel object (white porcelain cup). After 2 min, mice were placed back into the arena, and left to explore for 5 min.

#### T-maze spatial habituation

Exposure to a spatial location leads to habituation of exploration such that, in a novelty preference test, rodents subsequently prefer exploring a novel location to the familiar location. In the T-maze test of spatial habituation, each mouse was allowed to explore two arms of a T-maze made of acrylic sheet (0.6 mm thick) painted a light beige color. The main runway (65 × 14 × 30 cm) was connected to two side (goal) arms (30 × 14 × 30 cm). After 10 min (Cohort 1) or 1 hr (Cohort 2) of exposure training, a guillotine door was manually removed to reveal the third (novel) arm. The ambulation of the subject in each arm was then observed for 5 min. The exploration ratio was calculated as: “time in novel arm” divided by “time in familiar arms.”

#### Visible platform water maze

The visible platform version of the Morris water maze is a simple nonspatial learning task that is believed to be independent of hippocampal function (D’Hooge & De Deyn, 2001). The Morris water maze consisted of a cylindrical tub of ivory-colored acrylic sheet (117 cm diameter; 30 cm depth) that was filled with water (26 ± 1 °C temperature) to 11 cm below the rim, as described previously (Clapcote et al., 2005). The water was rendered opaque by the addition of white, nontoxic paint. A circular platform (10 cm diameter) made of transparent acrylic sheet was submerged 1 cm below the water surface at the center of the pool. The platform location was indicated by a high-contrast striped marker (1 cm diameter rod with a ping-pong ball affixed to the top) rising 13 cm above the water surface. Each subject was given four training trials. At the start of each trial, the mouse was placed by the tail into the water, immediately facing the perimeter, at one of the cardinal compass points (north, south, east, or west), and then was allowed a maximal time of 90 s to locate the platform. Finding the platform was defined as staying on it for at least 2 s.

#### Trace fear conditioning

Fear conditioning takes advantage of the natural tendency of rodents to freeze in response to fearful stimuli. Decreased freezing behavior is used as an indicator of a rodent’s recognition of a potentially aversive stimulus, and thus, an indicator of memory function. In trace conditioning, a conditioned stimulus (CS), such as an auditory tone, is presented. After a stimulus-free period called the trace interval, an unconditioned stimulus (US), such as a footshock, is presented. During the interval, the subject must sustain attention to learn a CS-US association. The fear conditioning apparatus used has been described previously (Kirshenbaum et al., 2013). For the training phase, mice were placed in the chamber for 2 min, after which the CS (auditory tone, 3,600 Hz, 95 dB) was presented for 30 s. There was then a 3-s interval before the US (continuous scrambled footshock, 1.0 mA) was presented for 2 s. Mice then remained in the chamber for an additional 30 s before being returned to their home cage. Twenty-four hours after training, the chamber was altered to evaluate freezing to the auditory tone. To disguise the chamber, the grid was covered with a smooth white acrylic sheet, the walls were altered by inserting a white acrylic sheet triangle, a 1% acetic acid odor was applied and the lights in the room were switched off. Subjects were placed in a chamber for 3 min to explore the new environment, followed by a 3 min presentation of the CS while freezing was recorded.

#### Social recognition

Social recognition is the ability to distinguish familiar from novel conspecifics. Social recognition by a subject mouse is defined by decreased investigation of a previously encountered mouse. The social recognition procedure was modified from a previously described protocol (Kogan, Franklandand, & Silva, 2000). To avoid potentially confounding effects of sexual behavior, only male mice were used for this experiment. On Day 1, training sessions consisted of a 15-min habituation to a new cage, followed by a 2-min (Cohort 3) or 10-min (Cohort 4) presentation of a novel juvenile male C57BL/6NCrl mouse (Charles River). Social investigation of the juvenile (mainly sniffing and licking of the anogenital region of the juvenile) by the adult mouse was observed continuously by an observer who scored the duration of investigation behavior with Observer software (Noldus). The social interaction time was measured, after which the mice were returned to their home cages. On Day 2, mice were tested 24 hr after the initial interaction using the same juvenile male. Any aggressive encounter between mice was immediate cause for terminating the experiment and excluding data from an adult from the analysis. The recognition ratio was calculated as: “social interaction time on Day 2” divided by “social interaction time on Day 1.”

#### Open field test of locomotor habituation

When placed in a novel environment, mice tend to explore for a period of time, and then reduce the level of exploration. This reduction in locomotor or exploratory behavior is known as habituation. Habituation to an open field arena made of clear acrylic sheet (41.25 × 41.25 × 31.25 cm) was measured as a decrement in exploratory locomotor activity over time. Mice were placed in the center of the floor, and allowed to freely explore the open field for 1 hr on each of four consecutive days. Locomotor activity was indexed by the total distance traveled (in meters) in each 1-hr session using the VersaMax Animal Activity Monitoring System (AccuScan Instruments).

### Statistical Analysis

Data were subjected to analysis of variance (ANOVA) to examine performance by genotype and sex as between-subjects factors. Tukey-Kramer post hoc multiple comparison tests were performed on any significant main effect or interaction. For each genotype in the object recognition test, one-way ANOVA was used to examine the effect of object location on the percentage of exploration time spent with each identical object. Student’s *t* tests were used in the Y-maze spontaneous alternation and object recognition tests to compare the performance of each genotype versus chance levels, in the trace fear conditioning test to compare freezing levels between various phases, in the social recognition test to compare the recognition ratio for each genotype versus a “null recognition” ratio of 1.0, and in the open field locomotor habituation test to compare between days. Significance level was set to 0.05. All values reported in the figures are expressed as mean ± *SEM*.

## Results

### Y-Maze Spontaneous Alternation

**Figure 1 fig1:**
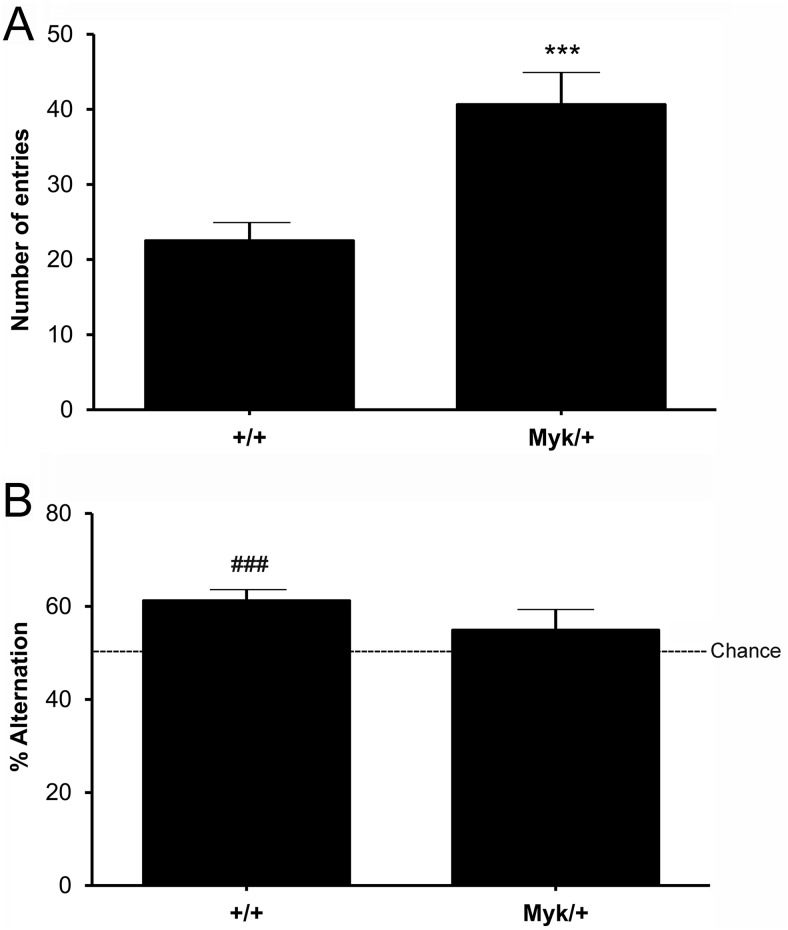
Y-maze spontaneous alternation. (A) Total number of arm entries (mean ± *SEM*) of *Myk*/+ (*n* = 10) and +/+ (*n* = 10) mice. (B) Percentage of alternation (mean ± *SEM*). *** *p* < .001 compared with +/+ mice. ^###^ *p* < .001 compared with chance level (50%).

In the Y-Maze spontaneous alternation test, all mice showed good ambulatory activity (above six entries within 5 min). A main effect of genotype was observed for number of arm entries, *F*(1, 16) = 12.33, *p* < .01, with no sex effect, *F*(1, 16) = 0.16, *ns* or sex by genotype interaction, *F*(1, 16) = 1.20, *ns* observed. *Myk*/+ mice had a greater total number of entries than +/+ mice (Figure 1A), indicating greater exploratory locomotion. No effects of genotype, *F*(1, 16) = 1.43, *ns*, sex, *F*(1, 16) = 0.05, *ns*, or genotype by sex interaction, *F*(1, 16) = 0.17, *ns* were observed for percentage of alternation. Although *Myk*/+ and +/+ mice showed roughly equivalent levels of alternation (Figure 1B), only +/+ mice significantly exceeded the chance level (+/+: *t*(9) = 4.73, *p* < .001; *Myk*/+: *t*(9) = 1.11, *ns*), suggesting impairment of *Myk*/+ mice in this measure of short term spatial memory.[Fig-anchor fig1]

### Object Recognition

**Figure 2 fig2:**
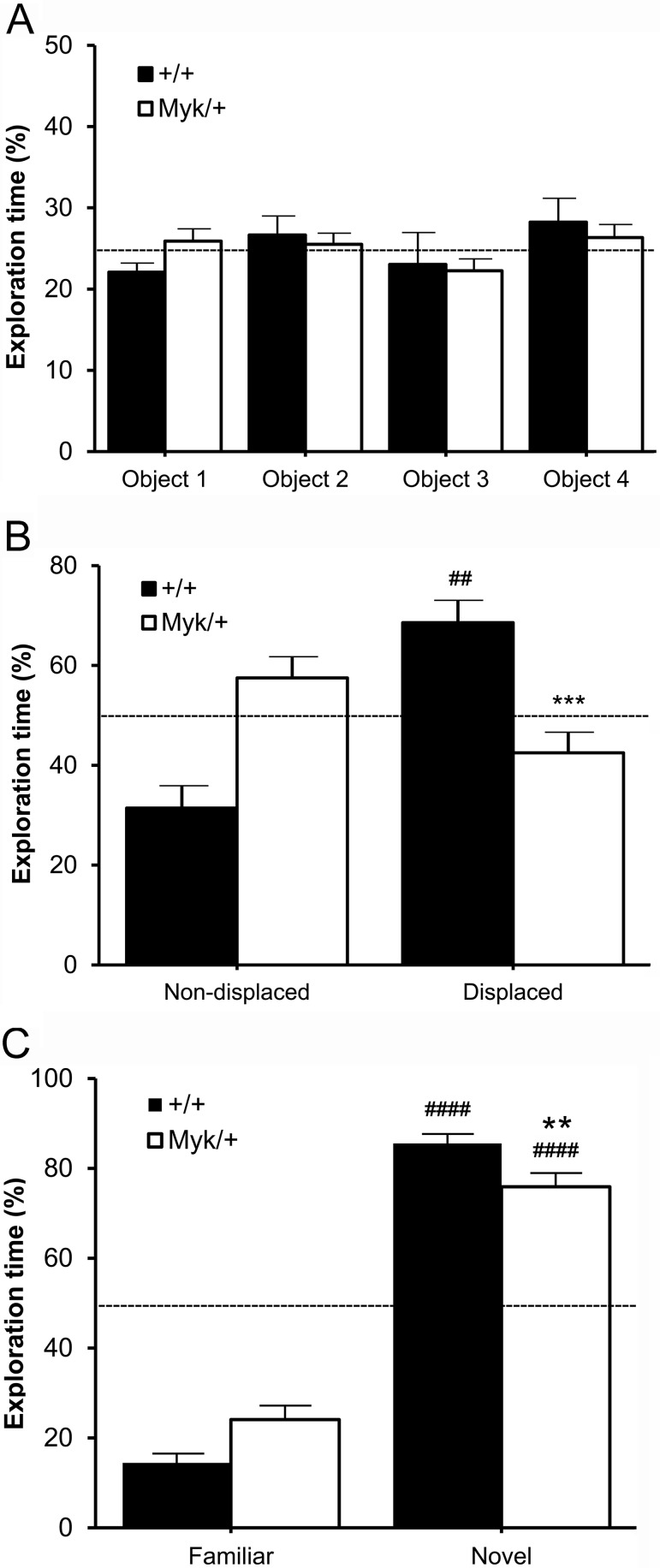
Object recognition. (A) Percentage of exploration time (mean ± *SEM*) spent by *Myk*/+ (*n* = 10) and +/+ (*n* = 10) mice exploring four identical objects. Broken line indicates 25% chance level. (B) Percentage of exploration time (mean ± *SEM*) with displaced objects versus nondisplaced objects. Broken line indicates 50% chance level. (C) Percentage of exploration time (mean ± *SEM*) with a novel object versus a familiar object. Broken line indicates 50% chance level. ** *p* < .01; *** *p* < .001 compared with +/+ mice. ^##^ *p* < .01; ^####^ *p* < .0001 compared with chance level (50%).

In the object recognition test, no effect of genotype, *F*(1, 16) = 0.05, *ns*, sex, *F*(1, 16) = 2.72, *ns* or sex by genotype interaction, *F*(1, 16) = 0.26, *ns* was observed for the total amount of time spent exploring all four objects. Within each genotype, no effect of object location was observed for the percentage of exploration time spent with each identical object (*Myk*/+: *F*(3, 36) = 1.65, *ns*; +/+: *F*(3, 36) = 1.13, *ns*; Figure 2A). After two of the objects were displaced from their original locations, no effect of genotype, *F*(1, 16) = 1.12, *ns*, sex, *F*(1, 16) = 2.46, *ns* or sex by genotype interaction, *F*(1, 16) = 0.23, *ns* was observed for the total amount of time spent exploring all four objects. However, a main effect of genotype was observed for the percentage of exploration time spent with the displaced objects, *F*(1, 16) = 17.35, *p* < .001, with no sex effect, *F*(1, 16) = 0.03, *ns* or sex by genotype interaction, *F*(1, 16) = 3.23, *ns* observed. *Myk*/+ mice showed a significant decrease in the percentage of exploration time spent with the displaced objects (Figure 2B). Only +/+ mice significantly exceeded the chance level (+/+: *t*(9) = 4.26, *p* < .01; *Myk*/+: *t*(9) = −1.79, *ns*). After three of the original objects were removed from the arena and replaced with a single novel object, no effect of genotype, *F*(1, 16) = 2.12, *ns*, sex, *F*(1, 16) = 1.05, *ns* or sex by genotype interaction, *F*(1, 16) = 0.60, *ns* was observed for the total amount of time spent exploring both objects. However, a main effect of genotype was observed for the percentage of exploration time spent with the novel object, *F*(1, 16) = 9.88, *p* < .01, with no sex effect, *F*(1, 16) = 3.61, *ns* or sex by genotype interaction, *F*(1, 16) = 0.467, *ns* observed. *Myk*/+ mice showed a significant decrease in the percentage of exploration time spent with the novel object (Figure 2C). However, both genotypes exceeded the chance level (+/+: *t*(9) = 17.22, *p* < .0001; *Myk*/+: *t*(9) = 8.22, *p* < .0001).[Fig-anchor fig2]

### T-Maze Spatial Habituation

**Figure 3 fig3:**
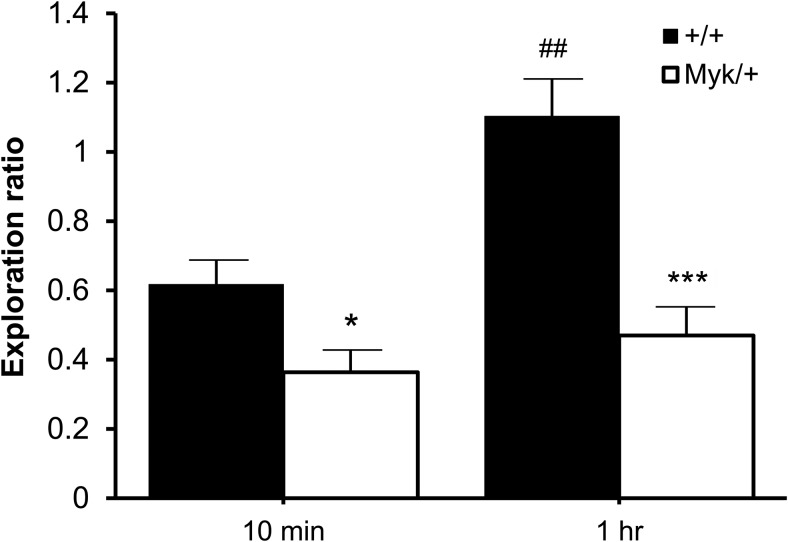
T-maze habituation. Exploration ratio (mean ± *SEM*) of *Myk*/+ (*n* = 10) and +/+ (*n* = 10) mice allowed to explore two arms of a T-maze for 10 min or 1 hr before the third (novel) arm was revealed. * *p* < .05; ** *p* < .01 compared with +/+ mice. ^##^ *p* < .01 compared with 10-min exploration ratio.

**Figure 4 fig4:**
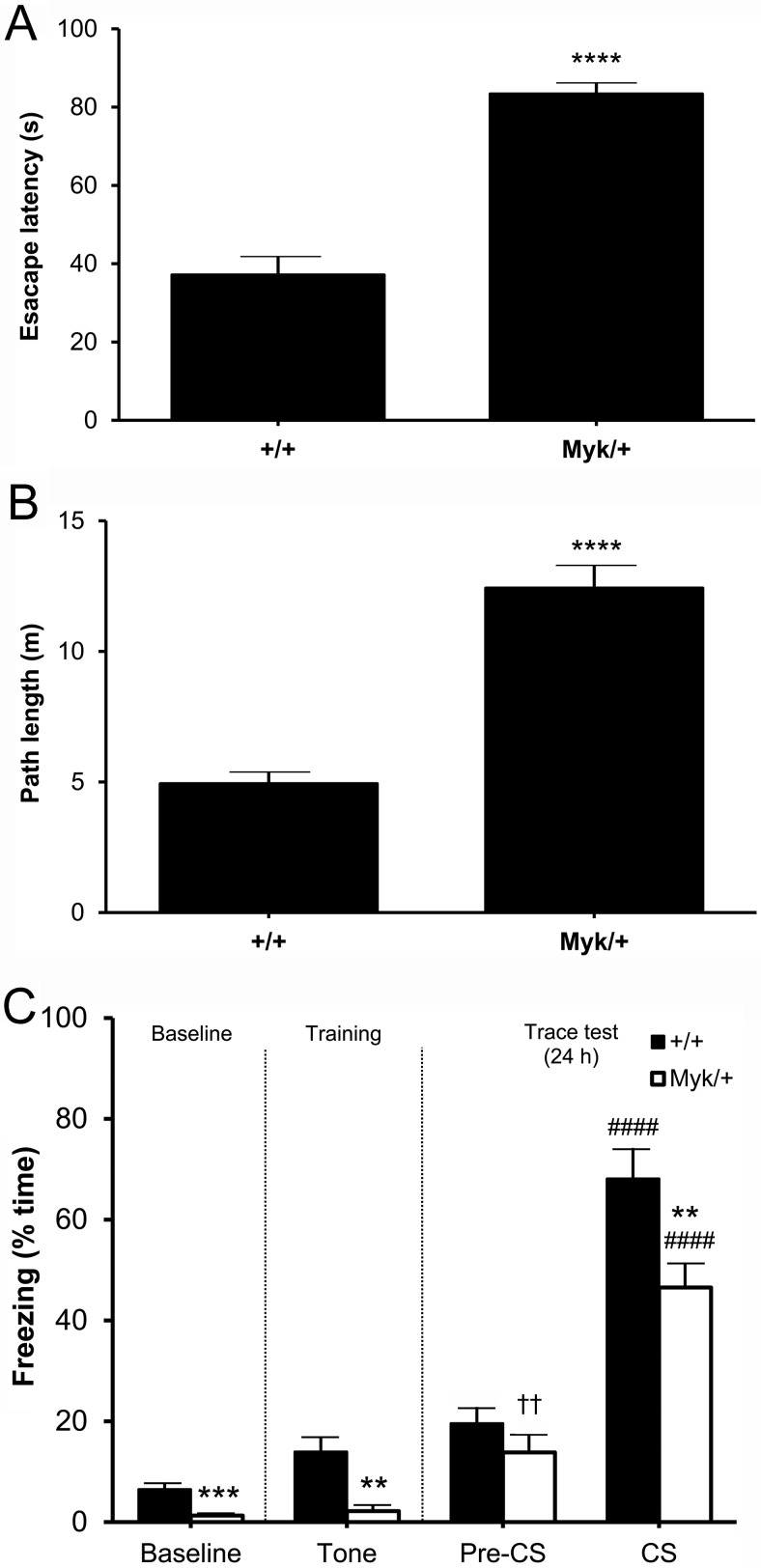
Water maze and fear conditioning. (A) Escape latency (s; mean ± *SEM*) and (B) swim path length (m; mean ± *SEM*) of *Myk*/+ (*n* = 10) and +/+ (*n* = 10) mice in the visible platform version of the Morris water maze test. (C) Freezing levels (mean ± *SEM*) of *Myk*/+ (*n* = 10) and +/+ (*n* = 10) mice in the trace fear conditioning procedure. Baseline, baseline (test-naïve) freezing; Tone, freezing during the auditory tone in the training phase; Pre-CS, freezing in the altered chamber before the auditory tone; CS, freezing in the altered chamber during the auditory tone. ** *p* < .01; *** *p* < .001 compared with +/+ mice. ^††^ *p* < .01 compared with Baseline. ^####^ *p* < .0001 compared with Pre-CS.

In the T-maze test of spatial habituation, Cohort 1 was allowed to explore two arms for 10 min, after which the third (novel) arm was revealed and the ambulation of mice in the novel versus familiar arms was observed for 5 min. A main effect of genotype was observed for 10-min exploration ratio, *F*(1, 16) = 6.10, *p* < .05, with no sex effect, *F*(1, 16) = 1.13, *ns* or sex by genotype interaction, *F*(1, 16) = 0.11, *ns* observed. With 10 min of exposure, *Myk*/+ mice spent proportionately less time in the novel arm than +/+ mice (see Figure 3). Cohort 2 was allowed to explore two arms for 1 hr before the third (novel) arm was revealed. A main effect of genotype was observed for 1-hr exploration ratio, *F*(1, 16) = 18.19, *p* < .001, with no sex effect, *F*(1, 16) = 0.11, *ns* or sex by genotype interaction, *F*(1, 16) = 0.17, *ns* observed. Even with 1 hr of exposure, *Myk*/+ mice still spent proportionately less time in the novel arm than +/+ mice (see Figure 3), indicating a deficit in spatial habituation. Only +/+ mice spent proportionately more time in the novel arm after 1 hr versus 10 min of exposure to the familiar arms (+/+: *t*(9) = −3.75, *p* < .01; *Myk*/+: *t*(9) = −1.00, *ns*; Figure 4).[Fig-anchor fig3][Fig-anchor fig4]

### Visible Platform Water Maze

Over four trials of up to 90 s duration, a main effect of genotype was observed for escape latency, *F*(1, 16) = 69.45, *p* < .0001 and path length, *F*(1, 16) = 48.20, *p* < .0001, with no sex effect (*F*(1, 16) < 0.10, *ns*) or sex by genotype interaction (*F*(1, 16) < 0.28, *ns*) observed. *Myk*/+ mice took more time (Figure 4A) and swam further (Figure 4B) than +/+ mice before reaching the visible platform. No effect of genotype (*F*(1, 16) < 3.94, *ns*), sex (*F*(1, 16) < 1.16, *ns*), or sex by genotype interaction (*F*(1, 16) < 1.50, *ns*) was observed for swim speed and floating, suggesting no gross impairment in the ability or motivation of *Myk*/+ mice to swim. However, a main effect of genotype was observed for thigmotaxis (wall-hugging), *F*(1, 16) = 8.30, *p* < .05, with no sex effect, *F*(1, 16) = 0.11, *ns* or sex by genotype interaction, *F*(1, 16) = 0.58, *ns* observed. Given the performance deficit of *Myk*/+ mice with a visible platform, we did not proceed to test them with a hidden platform.

### Trace Fear Conditioning

We previously observed that *Myk*/+ mice are impaired in delay fear conditioning (Kirshenbaum et al., 2013), in which the CS is immediately followed by the US. In trace conditioning, the end of the CS and the start of the US are separated by a trace interval. In this procedure, a main effect of genotype was observed for baseline (test-naïve) freezing, *F*(1, 16) = 17.39, *p* < .001 and freezing during the auditory tone (CS) in the training phase, *F*(1, 16) = 14.06, *p* < .01, with no sex effect (*F*(1, 16) < 1.52, *ns*), or sex by genotype interaction (*F*(1, 16) < 0.86, *ns*) observed. Both genotypes displayed baseline freezing of less than 10%, but that displayed by *Myk*/+ mice was significantly lower compared with +/+ mice (Figure 4C). Twenty-four hours after training, subjects were placed into an altered chamber and the CS was presented again. No effect of genotype, *F*(1, 16) = 2.13, *ns*, sex, *F*(1, 16) = 4.22, *ns* or sex by genotype interaction, *F*(1, 16) = 0.02, *ns* was observed for freezing in the altered chamber before the auditory tone (Pre-CS). Both genotypes increased their Pre-CS freezing relative to baseline freezing (+/+: *t*(9) = 3.02, *p* < .01; *Myk*/+: *t*(9) = 3.41, *p* < .01), suggesting that both genotypes showed some fear response to the altered chamber. During presentation of the auditory tone (CS), a main effect of genotype was observed for CS freezing, *F*(1, 16) = 10.17, *p* < .01, with no sex effect (*F*(1, 16) < 1.52, *ns*) or sex by genotype interaction (*F*(1, 16) < 0.86, *ns*) observed. Both genotypes increased their CS freezing level relative to Pre-CS freezing (+/+: *t*(9) = 10.29, *p* < .0001; *Myk*/+: *t*(9) = 10.84, *p* < .0001), although *Myk*/+ mice showed a significantly lower level of CS freezing compared with +/+ mice (Figure 4C).

### Social Recognition

**Figure 5 fig5:**
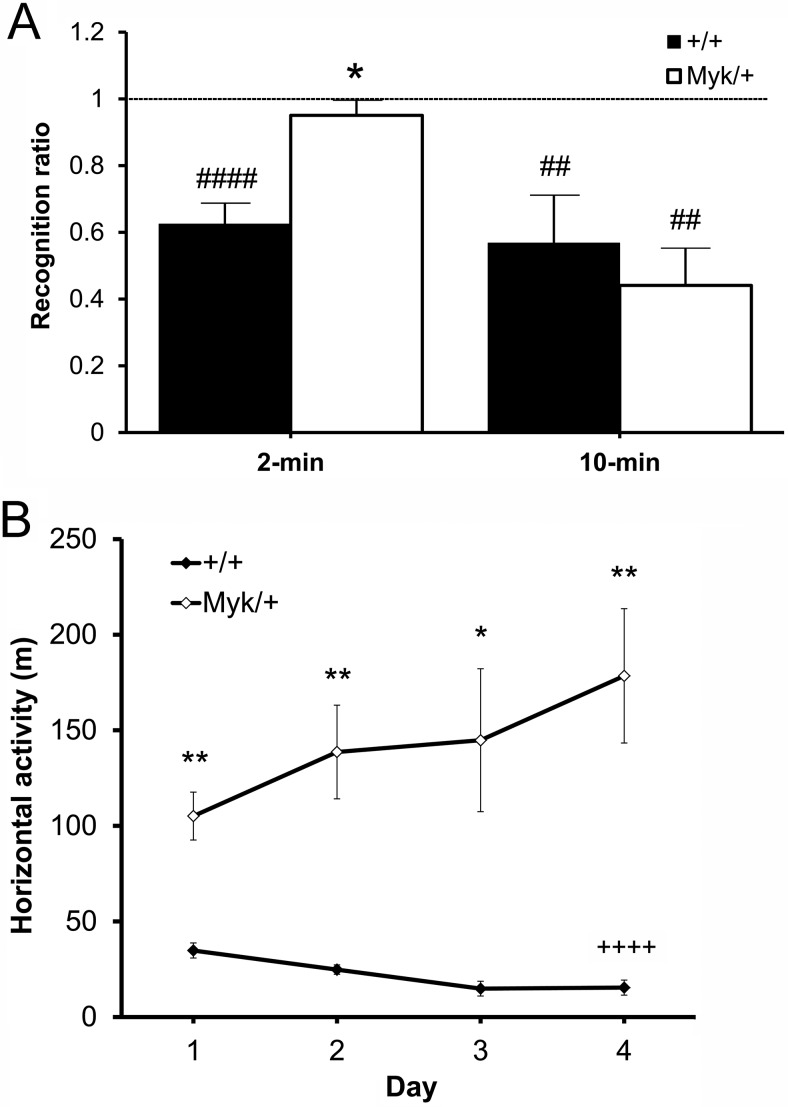
Social recognition and locomotor habituation. (A) Recognition ratio (mean ± *SEM*) of male *Myk*/+ and +/+ mice after exposure to a novel juvenile mouse for 2 min (*n* = 9/genotype) or 10 min (*n* = 6/genotype) the day before. Broken line indicates “null recognition” level (ratio of 1.0). (B) Total distance traveled (m) in an open field during each 1-hr session on four consecutive days. * *p* < .05 compared with +/+ mice. ^##^ *p* < .01; ^###^ *p* < .001 compared with null recognition level. ^++++^ *p* < .0001 compared with Day 1.

In the social recognition test, male *Myk*/+ and +/+ mice were exposed to a novel juvenile male mouse for 2 min (Cohort 3). A main effect of genotype was observed for the 2-min recognition ratio, *F*(1, 16) = 5.02, *p* < .05. Only +/+ mice had a 2-min ratio significantly below 1.0 (+/+: *t*(8) = −6.05, *p* < .001; *Myk*/+: *t*(8) = −0.34, *ns*). A separate cohort of male *Myk*/+ and +/+ mice (Cohort 4) was exposed to a novel juvenile male mouse for 10 min. No effect of genotype was observed for the 10-min recognition ratio, *F*(1, 10) = 0.74, *ns*. Both genotypes had 10-min ratios significantly below 1.0 (+/+: *t*(5) = −9.26, *p* < .001; *Myk*/+: *t*(5) = −4.88, *p* < .01; Figure 5A). This suggests that *Myk*/+ mice required a longer initial exposure time to recognize the pre-exposed juvenile.[Fig-anchor fig5]

### Locomotor Habituation in Open Field

Male *Myk*/+ and +/+ mice that had previously been tested in the social recognition test (Cohort 4) were subjected to the open field test of locomotor habituation. Main effects of genotype, *F*(1, 64) = 156.51, *p* < .0001, day, *F*(7, 64) = 2.60, *p* < .05 and genotype by day interaction, *F*(7, 64) = 3.71, *p* < .01 were observed for locomotor activity in each 1-hr session. The locomotor activity of *Myk*/+ mice was significantly greater than that of +/+ mice on all four test days (Figure 5B). The locomotor habituation deficit of *Myk*/+ mice, as suggested by the presence of a significant genotype by day interaction, was confirmed by the finding that only +/+ mice showed a significant decrement in locomotor activity between Day 1 and Day 4 (+/+: *t*(5) = −17.54, *p* < .0001; *Myk*/+: *t*(5) = 2.46, *ns*).

## Discussion

*Myk*/+ mice have an I810N amino acid substitution in Na^+^,K^+^-ATPase α3 that is identical to that present in AHC (Clapcote et al., 2009; Panagiotakaki et al., 2015; Yang et al., 2014). To investigate whether *Myk*/+ mice exhibit learning and memory deficits resembling the cognitive impairments of patients with *ATP1A3*-related disorders (Barbano et al., 2012; Paciorkowski et al., 2015, 2010; Sweney et al., 2009), we subjected them to a range of behavioral tests that interrogate various cognitive domains.

In the Y-maze spontaneous alternation test, *Myk*/+ mice showed increased exploratory locomotion, consistent with our previous observations in the hole-board test (Kirshenbaum, Clapcote et al., 2011). Although *Myk*/+ and +/+ mice showed roughly comparable levels of alternation, only +/+ mice exceeded the chance level, suggesting that *Myk*/+ mice were deficient in this measure of short term spatial memory. In the object recognition test, *Myk*/+ mice were significantly worse than +/+ mice at recognizing displaced objects, and did not perform above chance level. *Myk*/+ mice were better at recognizing novel objects (exceeding chance level in this task), but were still significantly worse than +/+ mice.

In the T-maze test of spatial habituation, *Myk*/+ mice showed a spatial habituation deficit after 10 min of training. Increasing the duration of training to 1 hr significantly increased the spatial habituation of +/+ mice, but had no effect on *Myk*/+ mice. Similarly, *Myk*/+ mice were deficient in the open field test of locomotor habituation. Unlike +/+ mice, they did not demonstrate locomotor habituation by reducing their exploratory activity over the 4 days of testing. Increasing the duration of training from 2 min to 10 min did, however, improve the performance of *Myk*/+ mice in the social recognition test. With 2 min of training, only +/+ mice demonstrated social recognition by showing less social interaction on Day 2 than on Day 1. When training was increased to 10 min, both genotypes demonstrated social recognition, indicating that *Myk*/+ mice required more training than +/+ mice.

In the Morris water maze, *Myk*/+ mice were impaired in navigating to a visible escape platform. Swim speed and duration of floating were normal, but thigmotaxis was significantly increased in *Myk*/+ mice. Increased thigmotaxis has previously been observed in mice with retinal degeneration (Clapcote et al., 2005a), but the normal head tracking of *Myk*/+ mice in an optokinetic drum suggests that their vision is not impaired (Kirshenbaum, Clapcote et al., 2011).

In the trace fear conditioning test, there was no genotypic difference in freezing in the altered chamber (Pre-CS freezing), but *Myk*/+ mice showed less freezing than +/+ mice to the auditory tone (CS freezing), suggesting an impairment in trace fear conditioning. The mean CS freezing level of 47% exhibited by *Myk*/+ mice in the trace fear conditioning test suggests that their ability to exhibit freezing was not grossly affected by their whole body tremor (Kirshenbaum et al., 2013). We previously reported that *Myk*/+ mice show a deficit in delay fear conditioning (Kirshenbaum et al., 2013), in which the CS is immediately followed by the US during training. This deficit was maintained regardless of whether the footshock (US) was 0.75 or 1.0 mA (Kirshenbaum et al., 2013).

We previously reported that *Myk*/+ mice show hyperambulation and a chaotic walking path in response to a novel open field (Kirshenbaum, Clapcote et al., 2011). This response to a novel environment is likely to have some influence on the performance of *Myk*/+ mice in the Y-maze, T-maze, open field, objection recognition and social recognition, as these tests are dependent on locomotor activity. It likely also explains the decreased baseline freezing of *Myk*/+ mice when put into the novel environment of the fear conditioning chamber. The apparent cognitive impairment of *Myk*/+ mice is likely not a mere consequence of their locomotor hyperactivity, though. We previously reported that *Myk*/+ mice show a deficit in conditioned taste avoidance (Kirshenbaum et al., 2013), a test of implicit memory that takes place in the home cage rather than a novel environment, and is not dependent on the locomotor activity or freezing of the subject.

Factor analysis permits recognition of clusters of strongly intercorrelated variables termed factors, and dissociation of them from other (minimally intercorrelated) factors. In an attempt to dissociate cognitive function from noncognitive performance characteristics, future work will apply factor analysis to a selection of variables known to best characterize the behavioral variability of *Myk*/+ and +/+ mice in several tests, as undertaken previously with prion protein-deficient mice (Valenti et al., 2001).

Although *Myk*/+ mice showed deficits in all of the cognitive tests in the present study, one form of learning is known to be intact in *Myk*/+ mice. We previously reported that a “genotype by day” interaction was not observed when we tested *Myk*/+ and +/+ mice on an accelerating rotarod on five consecutive days (Kirshenbaum et al., 2013). *Myk*/+ mice demonstrated intact motor learning, the ability to acquire new motor skills through practice. Rodents with lesions that selectively destroy the hippocampus are able to acquire a motor learning task, but show profound impairment in a novel object recognition memory task (Gould et al., 2002). Because young adult *Myk*/+ mice are known to exhibit medial temporal sclerosis and aberrant membranous whorls in hippocampal pyramidal neurons in CA3 and CA1 (Clapcote et al., 2009), the difference in the outcome of the rotarod versus the other cognitive tests used is likely because of the relative importance of various brain structures in each test.

Several rodent studies have used a pharmacological approach to perturb Na^+^,K^+^-ATPase function in the brain. Intracranial injection of ouabain, a specific Na^+^,K^+^-ATPase inhibitor that does not cross the blood–brain barrier (Sugimoto et al., 2011), has been shown to impair learning and memory in laboratory rats and mice (Mizumori et al., 1987; Sato et al., 2004; Tucker & Gibbs, 1979; Wang et al., 2013, 2014; Zhan et al., 2004). These studies all point to a relationship between inhibition of Na^+^,K^+^-ATPase in the brain and impairment of learning and memory, but they are limited by the similar affinities of the α2 and α3 subunits for ouabain (Sweadner, 1989); thus, precluding Na^+^,K^+^-ATPase α3 specificity.

Other mouse genetic studies provide support for the involvement of Na^+^,K^+^-ATPase α3 in memory formation. Heterozygous *Atp1a3*^D801N/+^ mice, which carry the most common mutation causing AHC (D801N), were deficient in a novel object recognition task and in the visible platform version of the Morris water maze (Hunanyan et al., 2015). Though they showed unimpaired contextual fear conditioning and motor learning on the accelerating rotarod (Hunanyan et al., 2015). Heterozygous *Atp1a3*^tm1Ling/+^ mice, which have a reduction of hippocampal α3 protein expression of around 60% and a reduction of total brain Na^+^,K^+^-ATPase activity (of α1, α2, and α3 combined) of around 16%, showed deficient spatial learning in the Morris water maze but normal memory in a novel object recognition task (Kirshenbaum, Saltzman et al., 2011; Moseley et al., 2007). After exposure to chronic variable stress, consisting of one or two unpredictable mild stressors per day for 6 weeks, *Atp1a3*^tm1Ling/+^ mice showed a greater reduction of total brain Na^+^,K^+^-ATPase activity (of around 33%) and a pronounced deficit in novel object recognition memory (Kirshenbaum, Saltzman et al., 2011).

An obvious difference between the published pharmacological and genetic approaches that perturb Na^+^,K^+^-ATPase function in vivo is that the genetic mutations are present from conception. While it is therefore possible that Na^+^,K^+^-ATPase α3 mutations could affect the nervous system throughout development, we previously reported that the α1 and α2 isoforms contribute more than α3 to total brain Na^+^,K^+^-ATPase activity in E18 fetuses, whereas α3 is the functionally dominant Na^+^,K^+^-ATPase isoform in the young adult brain (Clapcote et al., 2009). A difference in the functional importance of α3 versus the other Na^+^,K^+^-ATPase isoforms in utero and postnatally may explain why homozygous *Myk*/*Myk* pups all die perinatally, despite being born alive and appearing grossly normal at birth (Clapcote et al., 2009).

One factor that may contribute to the impaired cognition of *Myk*/+ mice is altered neuronal excitability. Within the CA1 stratum radiatum of the hippocampus, following θ burst stimulation (a plasticity induction protocol), the magnitude of the relationship between the population action potential to the field excitatory postsynaptic potential was increased compared with +/+ mice (Clapcote et al., 2009). Hence, after a high frequency train of stimuli, the CA3-CA1 pathway exhibits signs of hyperexcitability. It is possible that this aberrant synaptic activity is, in part, responsible for altered neural coding during behavior. Similar hyperexcitability within the dentate gyrus and CA3 caused by brain-derived neurotrophic factor overexpression in mice was associated with impaired performance in the passive avoidance test (Croll et al., 1999). However, the overall magnitude of long-term potentiation in *Myk*/+ mice is similar, albeit more variable, to that of +/+ mice (Clapcote et al., 2009), arguing against cognitive impairment being caused merely by a lack of synaptic plasticity. Further work is thus required to elucidate the link between hippocampal hyperexcitability and the cognitive deficits exhibited by *Myk*/+ mice.

Because *Myk*/+ mice showed deficits in all of the tests used in the present study, we conclude that they do exhibit learning and memory deficits resembling the broadly defined cognitive impairments of patients with *ATP1A3*-related disorders. A Chinese AHC patient with the same I810N mutation as *Myk*/+ mice is reported to have developmental delay (Yang et al., 2014), while a Belgian AHC patient with I810N has moderate intellectual disability and autism (Panagiotakaki et al., 2015). Specific deficits in intellectual, academic, memory, attention, and executive functioning exhibited by a child with AHC of unknown *ATP1A3* status have been reported (Shafer, Mayfield, & McDonald, 2005). Until similar comprehensive neuropsychological data on the I810N patients and other AHC patients with identified *ATP1A3* mutations become available, it would be unreliable to draw specific parallels with aspects of the *Myk*/+ cognitive phenotype at this stage. Nevertheless, by better defining the cognitive profile of *Myk*/+ mice, the present study has widened the range of AHC symptoms for which the *Myk*/+ model could be used as a tool in the identification of novel therapeutic strategies.
